# Urine-derived renal epithelial cells for deep phenotyping and transcriptomic response to therapy in Fabry disease

**DOI:** 10.1042/CS20255570

**Published:** 2025-07-28

**Authors:** Praveen Dhondurao Sudhindar, Sarah E. Orr, Eve Miller-Hodges, Elisa Molinari, Katrina Wood, Shalabh Srivastava, Colin G. Miles, Holly R. Mabillard, Zachary T. Sentell, Marco Trevisan-Herraz, Juliana E. Arcila-Galvis, John A. Sayer

**Affiliations:** 1 Translational and Clinical Research Institute, Faculty of Medical Sciences, Newcastle University, Central Parkway, Newcastle upon Tyne, United Kingdom; 2 Centre for Cardiovascular Science, Queen’s Medical Research Institute, University of Edinburgh, Edinburgh, United Kingdom; 3 Department of Cellular Pathology, Royal Victoria Infirmary Newcastle upon Tyne, Newcastle upon Tyne, United Kingdom; 4 Sunderland Royal Infirmary, Sunderland, United Kingdom; 5 Biosciences Institute, Faculty of Medical Sciences, Newcastle University, Newcastle upon Tyne, United Kingdom; 6 Renal Services, Newcastle Upon Tyne Hospitals NHS Foundation Trust, Newcastle upon Tyne, United Kingdom; 7 NIHR Newcastle Biomedical Research Centre, Newcastle upon Tyne, United Kingdom

**Keywords:** Fabry disease, RNA-seq, urine-derived renal epithelial cells, electron microscopy, chaperone, enzyme replacement therapy

## Abstract

Fabry disease is an X-linked lysosomal storage disorder caused by α-galactosidase A deficiency, leading to glycosphingolipid accumulation and progressive organ damage. Renal involvement is a major complication, yet diagnosis often requires an invasive kidney biopsy, and follow-up relies on indirect biomarkers or imaging, which lack specificity. Here, we present human urine-derived renal epithelial cells (hURECs) as a minimally invasive alternative for phenotyping renal Fabry disease and monitoring treatment response. Using hURECs from a newly diagnosed male Fabry disease patient, transmission electron microscopy (TEM) revealed lysosomal inclusions consistent with native kidney biopsy findings. Bulk RNA sequencing (RNA-seq) identified a transcriptomic disease signature, including dysregulated pathways involved in lipid metabolism homeostasis, ion transport, endoplasmic reticulum stress response, and collagen processing. Following systemic treatment of the patient with chaperone therapy, partial amelioration of the hUREC transcriptomic signature was observed during the first few months. However, by nine months, the signature began reverting toward baseline, correlating with continued kidney function decline. This prompted a transition to enzyme replacement therapy, with early evaluations showing transcriptomic stabilization. Our findings demonstrate that hURECs replicate key structural and molecular markers of renal Fabry disease and offer a non-invasive platform for longitudinal assessment of treatment response. TEM of hURECs provides a diagnostic alternative to biopsy, while RNA-seq-based transcriptomic profiling offers a sensitive and dynamic view of molecular changes, including key dysregulated pathways. This dual utility positions hURECs as a novel tool for improving the diagnosis, monitoring, and personalized management of kidney involvement in Fabry disease.

## Introduction

Lysosomes are acidic organelles, responsible for the degradation of biological molecules into their smaller components for the recycling of substrates. Lysosomes play a vitally important role in the degradation of components from phagocytosis, endocytosis, and autophagy. Lysosomal storage disorders (LSD) are characterized by errors in metabolism due to defective lysosomal function. Fabry disease is an X-linked LSD [1] caused by variants in *GLA* leading to a reduction or total loss of the α-galactosidase A enzyme. The cellular effect of α-galactosidase A enzyme deficiency is an accumulation of glycosphingolipids, mainly globotriaosylceramide (GL-3, also known as Gb3) inside cells [2], visible as intracellular inclusions known as ‘zebra bodies’ [3].

*GLA* encodes a 429-amino-acid precursor, which is processed to a 370-amino-acid glycoprotein, which carries out its function as a homodimer [2]. Over 400 *GLA* variants have been attributed to Fabry disease [2], most of which are unique to individual families and are spread across all seven coding exons [4]. The enzyme deficiency is not directly responsible for the disease phenotype; rather, it is the accumulated glycosylated substrates [5]. These accumulated glycosphingolipids lead to the initiation of pathological cascades [6] and the progression of Fabry disease. The clinical phenotypes of Fabry disease are complex, as each α-galactosidase A substrate is found in different concentrations in each cell type in the body; hence, each affected tissue in the body may have slightly varying disease mechanisms and outcomes, determined by cellular glycosphingolipid concentration and the function of each cell.

There are two major types of Fabry disease: classical and non-classical [7]. Male patients with classical Fabry disease have no residual α-galactosidase A function, while non-classical or atypical cases have low-level residual enzyme activity (typically 1–20%) [7]. For female patients, it is more complex as enzyme functionality cannot distinguish between the two forms [8], and diagnosis relies on genetic testing [9]. Fabry disease is a progressive disorder [6] and typically presents with cardiac, renal, and central nervous system features [10], as GL-3 accumulation mainly affects cardiomyocytes, renal epithelial cells, endothelial cells, and neurons of the autonomic nervous system [3].

Classical Fabry disease initially manifests in childhood, with significant early comorbidity [11], including gastrointestinal dysfunction, abnormal temperature tolerance, and pain crises, manifesting at an average age of 9 years in males and 16 years in females [12]. Without treatment, major cardiac and renal complications develop in early to mid-adult life [7]. The phenotype in females with Fabry disease is much more varied, ranging from asymptomatic to as severe as the male phenotype [9]. Around 70% of female carriers of *GLA* variants exhibit a Fabry disease phenotype, due to skewed X inactivation [7].

In comparison to classical Fabry disease, patients with atypical Fabry disease generally have a milder disease, with severe complications restricted to one organ system [7]. It is typically not detected until a patient presents with a complication, such as unexplained cardiomyopathy, stroke, or chronic kidney disease (CKD) [7].

The kidney complications of Fabry disease, observed in about 50% of males and 20% of females, include the accumulation of GL-3 within podocytes leading to podocyte injury, glomerulosclerosis, and proteinuria. Indeed, proteinuria is often one of the earliest signs of kidney involvement in Fabry disease and can range from mild to nephrotic levels and is an indicator of progressive kidney damage. GL-3 deposition in renal tubular cells and interstitial cells can lead to tubular atrophy and interstitial fibrosis [3], which are key contributors to CKD in Fabry disease patients. Related to tubular injury, Fabry disease patients may also exhibit impaired tubular reabsorption, leading to an inability to concentrate urine and electrolyte imbalances. The accumulation of GL-3 in endothelial cells of renal blood vessels can cause vascular dysfunction, further contributing to the progression of kidney damage. Hypertension is common in Fabry disease, often secondary to both glomerular and vascular involvement, and can further exacerbate CKD [13].

Fabry patients have been found to have a 12-fold increased risk of strokes and transient ischemic attacks when compared with the normal population, due to glycosphingolipid accumulation in cerebral blood vessels [14].

Previously, the incidence of Fabry disease was thought to be approximately 1 in 117,000 live male births [15]. However, newborn screening programs have suggested this value to be an underestimation [16]. The figure generally quoted for incidence of classical Fabry disease in males is 1 in 40,000 live births [17]. Atypical disease is more common than classical disease, with a ratio of atypical: classical of approximately 11:1 [18].

Currently, Fabry disease is incurable [8], though there are some specific treatment options which delay disease progression. These include enzyme replacement therapy (ERT) [19] and Galafold™ (Migalastat) treatment, which acts as a chaperone for certain missense variants [20].

ERT is the most common treatment for Fabry disease, and three different drugs are commercially available: Replagal (agalsidase alfa), Fabrazyme (agasidase beta) [8], and recently a next-generation ERT, Elfabrio (pegunigalsidase alfa). These treatments work by replacing the α-galactosidase A enzyme, thereby reducing the level of GL-3 accumulation. ERT treatments work best when they are started early in the disease course. The effect ERT has varies considerably based on the organ system investigated, with small but significant improvement in renal and cardiac function reported for ERT [8].

Chaperone therapy has also been approved for use in treating Fabry disease. Migalastat, commercially available as Galafold™, is a small-molecule imino sugar analog of the terminal galactose residue of GL-3, which binds reversibly to the active site of amenable α-galactosidase A enzymes [20]. This stabilizes the enzyme in the endoplasmic reticulum (ER) so that the enzyme is appropriately trafficked to the lysosome. The decreased pH in the lysosome (from neutral in ER to acidic in lysosome) and the availability of GL-3 cause Migalastat to dissociate from α-galactosidase A and be rapidly excreted from the cell. Migalastat is used to treat 35–50% of patients with amenable *GLA* missense variants [20] that lead to mutant proteins which are degraded due to reduced stability but have otherwise normal α-galactosidase A activity. Migalastat can be taken orally, which is more convenient than intravenous-based ERT. As a small molecule, it has an increased cellular and tissue distribution compared with ERT. A transgenic mouse model of Fabry disease has indicated that it may be able to cross the blood–brain barrier to treat the central nervous system manifestations of the disease [20]. Fabry disease is life-limiting if untreated. The average age of death for males is 58.2 years, while for females, it is 75.4 years [21].

Microscopy of urine samples from Fabry disease patients displays unique characteristics. Mulberry cells and mulberry bodies are uniquely found in the urine sediment of Fabry disease patients and are typical of the condition [22]. Mulberry cells are renal distal tubular epithelial cells with an accumulation of GL-3. They have a lamellar appearance, distinguishing them from fat bodies in the cell, when viewed under a phase-contrast microscope [22]. The amount of mulberry cells present in a patient urine sample may be associated with kidney disease progression [23]. The presence of a Maltese cross in urinary sediment can also feature in Fabry disease, although this feature is not unique [22].

Current standard biomarkers for Fabry disease-associated nephropathy only detect the late stages of kidney disease [24]. Commonly assessed kidney biomarkers are albuminuria, cystatin C, and serum creatinine-based estimated glomerular filtration rate (eGFR), kidney biopsies, and urine mulberry cells [24]. Most commonly, albuminuria is assessed to monitor kidney disease progression, though kidney damage can occur long before elevated albumin is detected in the urine [24]. Mulberry cells provide a non-invasive, quick, and cheap diagnostic test for Fabry disease and are already commonplace in diagnostics in Japan [22]; however, a lack of trained staff and availability of specialist phase-contrast microscopes reduces its utility in other areas. The only globally accepted reliable diagnostic method for confirming kidney involvement in Fabry is a kidney biopsy, followed by histological analysis and transmission electron microscopy (TEM) to detect zebra bodies [24]. These histological changes pre-date the onset of clinical symptoms, so a kidney biopsy may be useful where Fabry nephropathy is suspected.

RNA sequencing (RNA-seq) uses a cell population or a tissue sample to generate a gene expression profile in specific tissues [25]. This can be used to analyze signaling pathways which are differentially regulated under different conditions including kidney injury [26]. Various bioinformatic analyses may be carried out downstream to identify the specific genes or pathways that are the most or least highly expressed in the sample [27]. RNA-seq of urine-derived cells is an established research approach for defining disease signatures (for both diagnosis and prognosis) for human kidney diseases [28] but has not yet reached routine clinical practice. The small number of cells obtained from a single urine sample provides challenges for downstream applications.

To increase the yield from a urine sample, innovative approaches have been developed. Human urine-derived renal epithelial cells (hURECs) are renal tubular epithelial cells isolated from urine samples [29]. They are a useful resource and provide a much larger number of renal tubular cells to provide an insight into gene expression and cellular morphology in the kidney, without the need for invasive kidney biopsies. We have previously proven these to be useful when investigating kidney diseases and ciliopathies, such as Joubert syndrome [30] and nephronophthisis [31]. hURECs accurately portray pathogenic mechanisms in genetic kidney diseases such as Fabry disease [32] and have the potential to be useful in Fabry disease research, as GL-3 accumulates in renal tubular epithelial cells. As urine samples are readily available and non-invasive, hURECs have the potential to provide a large population of cells for downstream analysis of disease phenotypes, pathophysiological mechanisms, and response to therapies.

Here, we use Fabry patient urine samples to derive hURECs and show that they can be used as an alternative to a native kidney biopsy for diagnosis of renal Fabry disease using cellular phenotyping via TEM. Importantly, we show that a transcriptomic analysis of hURECs can recapitulate the Fabry nephropathy disease signature and can be used to monitor response to targeted therapies in individual patients with Fabry disease.

## Methods

### Clinical and genomic studies

Genetic, clinical, and *in vitro* investigations were carried out on a patient with a recent diagnosis of Fabry disease. Clinical, imaging, and renal histological data and family information were obtained by reviewing clinical records. The collection of clinical data was undertaken with informed consent, and ethical approval was given by the National Research Ethics Service Committee Northeast reference 14/NE/1076.

### Histological stains

For hematoxylin and eosin staining, the slides containing the percutaneous renal biopsy tissue sections were deparaffinized and stained with Mayer’s hematoxylin for 1 min. Slides were rinsed with tap water and then washed three times with distilled water. The slides were stained with alcoholic eosin for 1 min and then, without rinsing the slides, were dehydrated through three changes of 95% ethanol and two changes of 100% ethanol for 1 min each. Finally, the slides were cleared in three changes of xylene before mounting and imaging.

### hUREC culture

The hURECs were isolated from urine collected from the Fabry disease patient (male) and two age and sex-matched controls (also male). The hURECs were cultured as previously described [29,30]. This methodology takes advantage of the fact that renal epithelial cells are regularly sloughed off from the nephron/renal tubules and end up in the urine. These epithelial cells can be collected from the urine and specifically cultured to support proliferation of renal epithelial cells, while suppressing the growth of other cell types present in the urine (e.g. transitional and squamous cells). Approximately 50–150 ml of mid-stream urine (from a void that is not the first of the day) is collected inside of a sterile container, and the samples are processed immediately or stored at 4°C for up to 4 h, which allows transporting samples to the laboratory. Then, the fresh urine samples were centrifuged at 400 g for 10 min to form a urinary sediment pellet. The pellets were washed with phosphate-buffered saline containing antibiotics to minimize the risk of contamination and resuspended in primary medium [DMEM F12, 10% Fetal Bovine Serum (FBS), Penicillin/Streptomycin, amphotericin B, renal epithelium cell growth medium (REGM) SingleQuots (Lonza, CC-4127)] in 12-well plates. After 72 h, the medium was replaced by proliferation hUREC medium [renal epithelium basal medium (REBM; Lonza, CC-3191), REGM SingleQuots, and 2% FBS]. A subset of cultured hURECs was treated *in vitro* with 100 μM of Migalastat (Galafold™; Merck, #D9641) for 24 h to allow its chaperone activity to be tested [33].

### Transmission electron microscopy

The hURECs were fixed for TEM in 1 ml of 2% Glutaraldehyde in 0.1 M sodium cacodylate buffer at pH 7.4 following two centrifugations at 200 g for 5 min. The fixed cells were refrigerated before imaging by the Electron Microscopy Research Services at Newcastle University using a Hitachi HT7800 120kV TEM with cryo-TEM capabilities.

### hURECs RNA-seq

At 90% confluence, the hURECs were dissociated using TrypLE (GIBCO, 12604013). The cell pellets were washed once with PBS and frozen at –80°C and sent to Novogene commercial facility for RNA extraction and RNA-seq. RNA-seq experiments were performed in biological triplicates at baseline for patient and control and one replicate over treatment time course.

### Data preprocessing and quality control

Raw sequencing reads were processed by Novogene (Novogene Co., Ltd., U.K), which included quality trimming, removal of adapters, and alignment to the human reference genome (GRCh38). Clean reads were mapped to the reference genome using HISAT2 version 2.0.5 with default parameters to generate BAM files. Quality control of the processed reads was independently verified using FastQC version 0.12.1 [34].

Data analysis was performed using R version 4.4.0 (R Core Team, 2013). Gene expression quantification was performed using the summarizeOverlaps() function from the GenomicAlignments package version 1.42.0. This function counts the number of reads overlapping with exons grouped by gene. To minimize biases due to cell-type heterogeneity in urine-derived samples, only genes with counts greater than zero in more than two samples were included in the analysis. Additionally, only genes with more than ten counts per sample were retained for analysis.

Data manipulation was performed using dplyr version 1.1.4 [35], tidyr version 1.3.1 [36], and reshape2 version 1.9.6 [37]. Counts per million (CPM) were calculated using the edgeR package version 4.2.2 [38], and principal component analysis (PCA) was performed on the log-transformed count data (log2 CPM + 1) using the prcomp() function in R. The scale. = TRUE parameter was used for data scaling. The top two principal components (PC1 and PC2) were extracted and visualized in a scatter plot using ggplot2 version 3.5.1 [39].

### Differential expression analysis

Differential expression analysis was carried out using DESeq2 version 1.44.0 [40]. Genes with an adjusted *P*-value (padj) <0.05 and absolute log2 fold change (log2FC) >1 were considered differentially expressed. Up-regulated and down-regulated genes were identified. A volcano plot was generated using the ggplot2 package version 3.5.1 in R [39].

### Functional enrichment analysis

Pathway enrichment analysis was performed using gene set enrichment analysis [41], using the package fgsea package on R, version 1.24.0 [42]. Genes were ranked based on test statistic (column ‘stat’ in DESeq2’s output, as recommended by the authors of DESeq2). The results were summarized using the aPEAR package version 1.0.0 [43], and a volcano plot was generated to visualize the expression profiles of the top significant genes with the smallest log fold change error rate (LFSE) values for relevant using the R package pheatmap version 1.0.12 [44].

### Linear regression analysis

Linear regression analyses were conducted in R to evaluate the trend of eGFR decline across three phases: pre-treatment, chaperone treatment, and ERT. Regression models were fitted using the lm() function from the stats package (version 3.6.2). The slopes of the fitted regression lines were calculated for each phase to determine the rate of eGFR decline. Scatterplots of the regression results were plotted using the ggplot2 package version 3.5.1 [39].

### Hierarchical clustering analysis

To assess whether the Fabry disease hUREC samples over time, during treatment with chaperone or ERT, grouped similarly to the baseline (untreated) or control groups, hierarchical clustering of the samples was performed using the hclust() function from the stats package in R [45]. The dissimilarity between samples was calculated, and the complete linkage method was applied to determine the dissimilarity between clusters based on the largest distance between any two samples within the clusters. A dendrogram was drawn to visualize the clustering structure, allowing for an assessment of the grouping behavior of the samples relative to the baseline and control groups.

### Gene expression-based similarity score

#### Z-score normalization

For each gene, expression values were z-score normalized across all samples to standardize expression distributions:


$$Z_{g,s}=\frac{zCPM_{g,s}-\mu_{g}}{\sigma_{g}}$$

where ${CPM}_{g,s}$ is the CPM expression value of gene *g* in sample *s,* $Z_{g,s}$ is the z-scored expression of gene *g* in sample *s*, and $\mu_{g}$ and $\sigma_{g}$ are the mean and standard deviation of CPM values for gene *g* across all samples.

#### Gene-level similarity score

For each gene *g* and treated sample *s*, we calculated the similarity score as:


$${Score}_{g,s}=|Z_{g,s}-Z_{g,baseline}|-|Z_{g,s}-Z_{g,control}|$$

where $Z_{g,s}$ is the z-score of gene *g* in the treated sample *s*, $Z_{g,baseline}$ is the average z-score across baseline (pre-treatment) samples, and $Z_{g,control}$ is the average z-score across control (healthy) samples.

A positive score indicates that the gene’s expression in the treated sample is more similar to the control condition than to the baseline. In contrast, a negative score reflects closer resemblance to the baseline state. Scores that are near zero imply that the expression level is approximately equidistant from both control and baseline states and may represent ambiguous or intermediate changes in expression.

#### Composite similarity score

To summarize gene-level responses into a sample-level metric, we computed the average score across all N genes:


$${CompositeScore}_{s}=\left(\frac{1}{N}\right)\times \sum_{g=1}^{N}{Score}_{g,s}$$

where ${CompositeScore}_{s}$ is the similarity score for sample*s*, and *N* is the total number of genes in the gene set.

This score provides a single interpretable metric per sample, where higher values indicate closer similarity to a healthy transcriptional state, lower values indicate persistence of a diseased profile, and intermediate values suggest partial transcriptional recovery.

## Results

### Diagnosis and molecular confirmation of Fabry disease with renal involvement in a male adult patient

**Figure 1:**
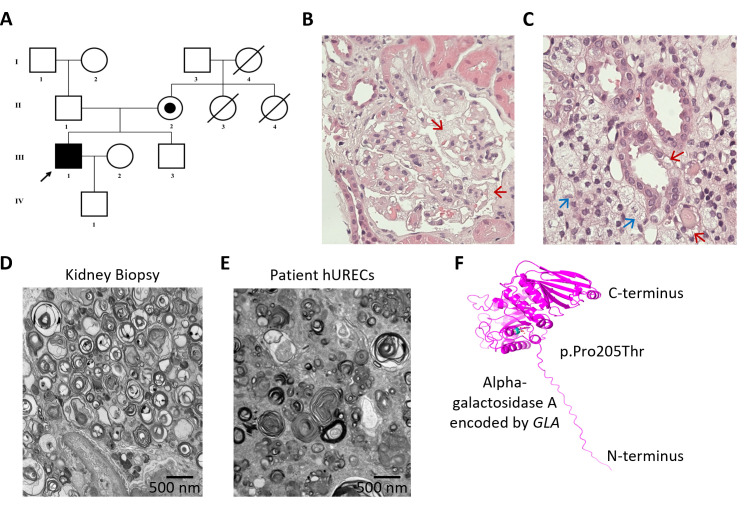
Clinical, histological, and genetic analysis of a patient with Fabry nephropathy. (**A**) Family pedigree diagram showing the proband (III:1) diagnosed with Fabry disease (indicated by an arrow). The pathogenic *GLA* variant was inherited from his mother (II:2). (**B, C**) Renal biopsy from the proband. (**B**) Light microscopy of a hematoxylin and eosin-stained section (×400) showing a glomerulus with podocytes demonstrating swollen cytoplasm containing fine vacuolation (red arrows). (**C**) Renal tubular epithelial cells exhibit a foamy appearance (red arrows) with interstitial foam cells also present (blue arrows) (haematoxylin and eosin ×400). (**D**) Electron microscopy (EM) image of the renal biopsy showing the presence of typical osmiophilic lamellated inclusions and zebra bodies. Image taken at 5000× magnification; scale bar = 500 nm. (**E**) EM image of human urine-derived renal epithelial cells (hURECs) from the proband, showing lamellated inclusions/zebra bodies comparable to those observed in the native renal biopsy. Image taken at 5000× magnification; scale bar = 500 nm. (**F**) Predicted three-dimensional structural model of human α-galactosidase A, encoded by the *GLA* gene on chromosome X. The structure was generated using the AlphaFold Protein Structure Database (https://alphafold.ebi.ac.uk) and UniProtKB (https://www.uniprot.org/uniprot/) with associated codes AF-P06280-F1v4 and P06280, respectively. The position of the missense single-nucleotide variant c.613C>A (p.Pro205Thr) is highlighted in cyan. Image generated using PyMOL.

The index patient (Figure 1A) was diagnosed with Fabry disease aged 30–35 years, after presenting with severe abdominal pain. A blood test at presentation had revealed elevated serum creatinine levels, and urine tests revealed significant proteinuria. A kidney biopsy was performed, which revealed typical features of renal Fabry disease under light and TEM (Figure 1B–D). Plasma α-galactosidase A levels were found to be very low [<0.4 nmol/h/ml (normal range 4.00–21.90)] in keeping with classical Fabry disease. Molecular genetic confirmation of Fabry disease was obtained by directly sequencing *GLA*, revealing a known hemizygous pathogenic missense variant [NM_000169.3; c.613C>A; p.(Pro205Thr)] (Figure 1F), and familial segregation studies revealed that he had inherited this variant from his mother (Figure 1A).

α-Galactosidase A is a homodimer with each monomer containing distinct structural regions, an N-terminal catalytic domain and a C-terminal β-sandwich domain. The N-terminal catalytic domain contains a (β/α)₈ barrel (triose-phosphate isomerase (TIM) barrel) fold, which is a common feature of many glycoside hydrolases. The C-terminal β-sandwich domain is composed of antiparallel β-strands; this domain likely contributes to structural stability [46]. The active site resides in the N-terminal domain within the (β/α)₈ barrel. There are two key residues that play critical roles in the catalytic mechanism of this region: Asp170 (participates in the cleavage of the glycosidic bond by forming a covalent glycosyl-enzyme intermediate) and Asp231, which facilitates proton transfer necessary for glycosidic bond cleavage [47].

Key structural features of the protein include three glycosylation sites (N139, N192, and N215), which are crucial for stability, lysosomal targeting, and activity. Furthermore, the substrate binding pocket, whereby α-galactosidase A selectively binds α-linked galactose residues, is crucial for its enzyme activity [48].The amino acid Pro205 has proximity to the active site, and this region contributes to maintaining the proper geometry of the active site and substrate binding pocket (Supplementary Figure S1).

Urine samples were obtained from the patient while he was treatment-naïve (and then subsequently during his treatment course). From these urine samples, hURECs were successfully cultured, and TEM was used to assess the level of sphingolipid accumulation, which revealed a large number of osmiophilic lamellated myelin-like inclusions and zebra bodies (Figure 1E), as seen in his native renal biopsy (Figure 1D), and consistent with his diagnosis of Fabry nephropathy.

### Differentially expressed genes and pathways in Fabry disease

To better understand the molecular mechanisms involved in Fabry nephropathy, we assessed changes in gene expression between the Fabry patient and two healthy controls. To this end, we collected urine samples from the Fabry patient at three different time points pre-treatment (t0, *r*=3), and two samples at different time points from a healthy individual (Control 1, *r*=2) and one sample from a second healthy individual (Control 2, *r*=2). All samples were morning urine samples, second void of the day for patient and healthy controls. The subsequent follow-up time points were determined by patient clinic visits and their scheduled treatment follow-up. Healthy control samples were collected at the same time and day as patient samples. The hURECs were obtained from these urine samples. RNA was extracted from these cultures and sent for bulk RNA-seq (Supplementary Figure S2).

Prior to differential expression analysis, we used the RNA-seq data to assess the expression of selected genes that serve as markers for specific cell types from different parts of the nephron. This was performed because hURECs are heterogeneous, and we wanted to confirm that the representation of kidney cell types in the Fabry patient and control samples was similar. We found comparable expression levels of these markers between the Fabry patient and control samples, indicating no significant bias in cell-type representation (Supplementary Figure S3).

**Figure 2:**
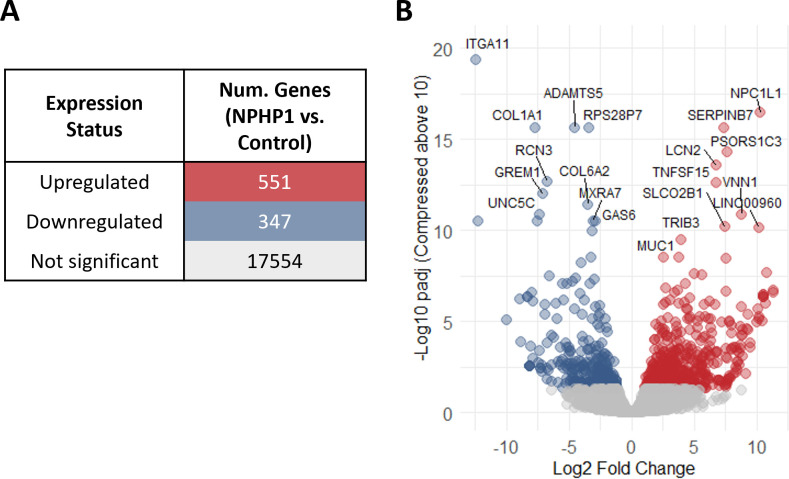
Gene expression analysis comparing Fabry vs. control. (**A**) Table summarizing the number of genes categorized as up-regulated, down-regulated, and not Significant based on differential expression analysis (threshold of |Log2FC| >1 and padj <0.05). (**B**) Volcano plot showing the differential gene expression distribution, where genes with significant up-regulation are shown in red, down-regulation in blue, and non-significant genes in gray. To enhance visualization, values greater than 15 on the y-axis have been log-transformed using the following formula: log-transformed y_value = 15+log_2_(y_value−14).

After analysis of differential gene expression, we identified 551 up-regulated genes with a log2FC greater than 1 and a padj less than 0.05, and 347 down-regulated genes with a log2FC less than 1 and padj less than 0.05 (Figure 2, Supplementary Table S1). The most significantly up-regulated genes included lipid transport-related genes such as *NPC1L1* (log2FC = 10.3, padj = 1.9×10^−17^), *LCN2* (log2FC = 6.8, padj = 2.8×10^−14^), and inflammation-related genes such as the cytokine *TNFSF15* (log2FC = 6.8, padj = 2.4×10^−13^) and the membrane-bound protein *MUC1* (log2FC = 3.8, padj = 3×10^−9^). Up-regulation of *MUC1* is associated with renal tubular injury and fibrosis. Additionally, we observed the up-regulation of *VNN1*, a gene associated with renal tubular injury and cell senescence (log2FC = 8.8, padj = 1.3×10^−11^). Other noteworthy up-regulated genes included two long non-coding RNAs (lncRNAs): *PSORPS1CR* (log2FC = 7.7, padj = 5.5×10^−15^), which is associated with psoriasis and thought to enhance the expression of the transcription factor OCT4 (known for regulating stemness, tumorigenesis, and stress responses), and the long non-coding RNA gene *LINCOO960* (log2FC = 10.2, padj = 7.1×10^−11^), involved in the regulation of cell proliferation and glycolysis. Furthermore, *TRIB3* (log2FC = 3.9, padj = 3.3×10^−10^), involved in the palmitate-induced apoptosis pathway, and *SERPINB7* (log2FC = 7.4, padj = 2.9×10^−16^), a serine proteinase inhibitor up-regulated in IgA nephropathy and thought to contribute to mesangial matrix accumulation by decreasing matrix metalloproteinase activities, were also significantly up-regulated (Figure 2).

Among the down-regulated genes, we found several extracellular matrix (ECM)-related genes, including *ITGA11* (log2FC = −12.5, padj = 9.4×10^−35^), which is part of the integrin pathway, as well as *COL1A1* (log2FC = −7.7, padj = 2.9×10^−16^), encoding collagen type 1, and *COL6A2* (log2FC = −3.5, padj = 3.7×10^−12^), which encodes collagen type 6. Additionally, we observed down-regulation of the metalloproteinase *ADAMTS5* (log2FC = −4.5, padj = 2.9×10^−16^), a key enzyme in collagen degradation, as well as *RCN3* (log2FC = −6.7, padj = 2.1×10^−13^), a negative regulator of collagen type 3 production, and *MXRA7* (log2FC = −3.1, padj = 3.1×^10−11^), a matrix remodeller. Furthermore, we found the down-regulation of *GREM1* (log2FC = −7.1, padj = 9.8×10^−13^), a negative regulator of bone morphogenetic proteins (BMP) signaling through the transforming growth factor beta (TGF-β) pathway, and *GAS6* (log2FC = −2.8, padj = 3.12×10^−11^), a mitogen involved in regulating cell proliferation and survival. We also identified the down-regulation of *UNC5C* (log2FC = −7.3, padj = 1.3×10^−11^), a tumor suppressor gene that directs cell migration and axon extension, as well as the pseudogene *RPS28P7* (log2FC = −3.4, padj = 2.91×10^−16^) (Figure 2).

We performed a pathway enrichment analysis using gene ontology (GO), identifying 183 pathways significantly enriched in dysregulated genes. Among the pathways enriched in down-regulated genes, we found collagen fibril organization [GO:0030199, normalized enrichment score (NES) = −3.03, padj = 0.01], ECM organization (GO:0030198, NES = −2.96, padj = 0.01), mitotic cell cycle process (GO:1903047, NES = −2.75, padj=0.01), and cytoskeleton organization (GO:0007010, NES = −2.21, padj = 0.01). In contrast, the pathways enriched in up-regulated genes included transmembrane transport (GO:0055085, NES = 2.05, padj = 0.01), cholesterol homeostasis (GO:0042632, NES = 2.11, padj = 0.01), hexose metabolic process (GO:0019318, NES = 2.00, padj = 0.02), response to stress (GO:0006950, NES = 1.60, padj = 0.03), and calcium ion homeostasis (GO:0055074, NES = 1.89, padj = 0.036) (Supplementary Table S2).

**Figure 3:**
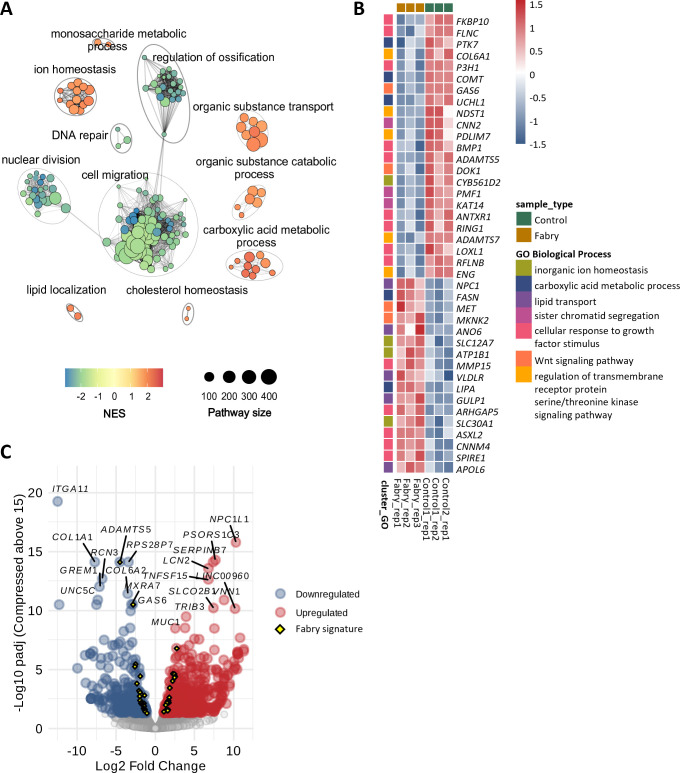
Pathway enrichment and expression profiles of genes involved in Fabry disease. (**A**) GO term Enrichment Analysis. Each node represents a GO term associated with a biological process, coloured by the normalized enrichment score (NES), where higher scores indicate significant enrichment of genes in the respective terms—positive NES reflects up-regulated genes, while negative NES indicates down-regulated genes. Links between nodes signify similarity measured as overlap in gene content. Clusters of similar pathways are delineated by ellipses, highlighting redundant or overlapping processes. The size of each node corresponds to the number of genes associated with that GO term, specified because enrichment analyses are especially sensitive to gene set size, with a propensity toward detecting larger gene sets as significant. (**B**) The heatmap displays the expression profiles of selected genes with a log fold change error rate (LFSE) <0.5, which are significantly dysregulated in Fabry patients. These genes belong to specific biological processes identified in the clusters shown in panel A. Annotations are provided to indicate the biological processes associated with each gene. (**C**) Overlay of the differential expression analysis shown in Figure 2B, now annotated to emphasize the genes displayed in panel B (highlighted as yellow diamonds). This view contextualizes their statistical significance and direction of change within the global transcriptomic profile.

We summarized the results using a clustering approach to group pathways based on gene content overlap, which helped identify the main biological processes affected (Figure 3). These processes included monosaccharide metabolic processes, which are consistent with GLA enzyme deficiency in Fabry disease. GLA is essential for the breakdown of GL-3, a process that normally involves the cleavage of galactose from ceramide [49]. This deficiency impairs the normal turnover of galactose, affecting monosaccharide metabolism. We also observed enrichment in pathways related to organic transport and catabolism, as well as lipid localization and cholesterol homeostasis. This is consistent with the accumulation of GL-3, which causes stress in the ER, a key organelle responsible for lipid biosynthesis and processing. ER dysfunction can affect pathways involved in organic transport and catabolism, which are responsible for managing lipid buildup. Additionally, we found enrichment in ion homeostasis-related pathways, which is consistent with the ER’s critical role in the synthesis, trafficking, and regulation of transport proteins [50].

We then identified the genes involved in the specific pathways within the clusters and selected those genes with an LFSE less than 0.5 to avoid spurious or biased results, considering our small sample size (*r*=3 cases and *r*=3 controls). This gene set included 17 up-regulated genes and 23 down-regulated genes (Figure 3). Among the up-regulated genes, several lipid homeostasis-related genes were identified, such as *VLDLR* (very low-density lipoprotein receptor, which is involved in the uptake of lipoproteins, and *LIPA*, which encodes lysosomal acid lipase, a key enzyme in lipid metabolism and *FASN*, which encodes fatty acid synthase, was also up-regulated. FASN is a key enzyme in fatty acid biosynthesis, playing a central role in lipid production and metabolism. Additionally, we found matrix-related genes like *MMP15*, which encodes matrix metalloproteinase 15, involved in ECM remodeling. We also identified *SLC12A7*, which encodes a solute carrier involved in ion transport, and *MET*, which encodes a receptor tyrosine kinase involved in cell survival and growth. Among the down-regulated genes, we identified *COL6A1*, which encodes collagen type 6 alpha 1, a key structural component of the ECM in connective tissue, and *FKBP10*, which is involved in collagen folding and assembly. The gene *LOXL1* encodes the lysyl oxidase-like 1, which is involved in collagen and elastin cross-linking and may affect ECM integrity.

### Temporal changes in kidney function, zebra bodies, and gene expression during chaperone treatment and transition to enzyme replacement therapy in Fabry patients

Over the next 13 months, the patient’s eGFR dropped from 42 ml/min/1.73 m² to 30 ml/min/1.73 m², and treatment was switched from Migalastat (Galafold™) to ERT. Follow-up assessments, including urine sample collection, hUREC culture, RNA-seq, and TEM, were conducted during the treatments (Supplementary Figure S4).

**Figure 4:**
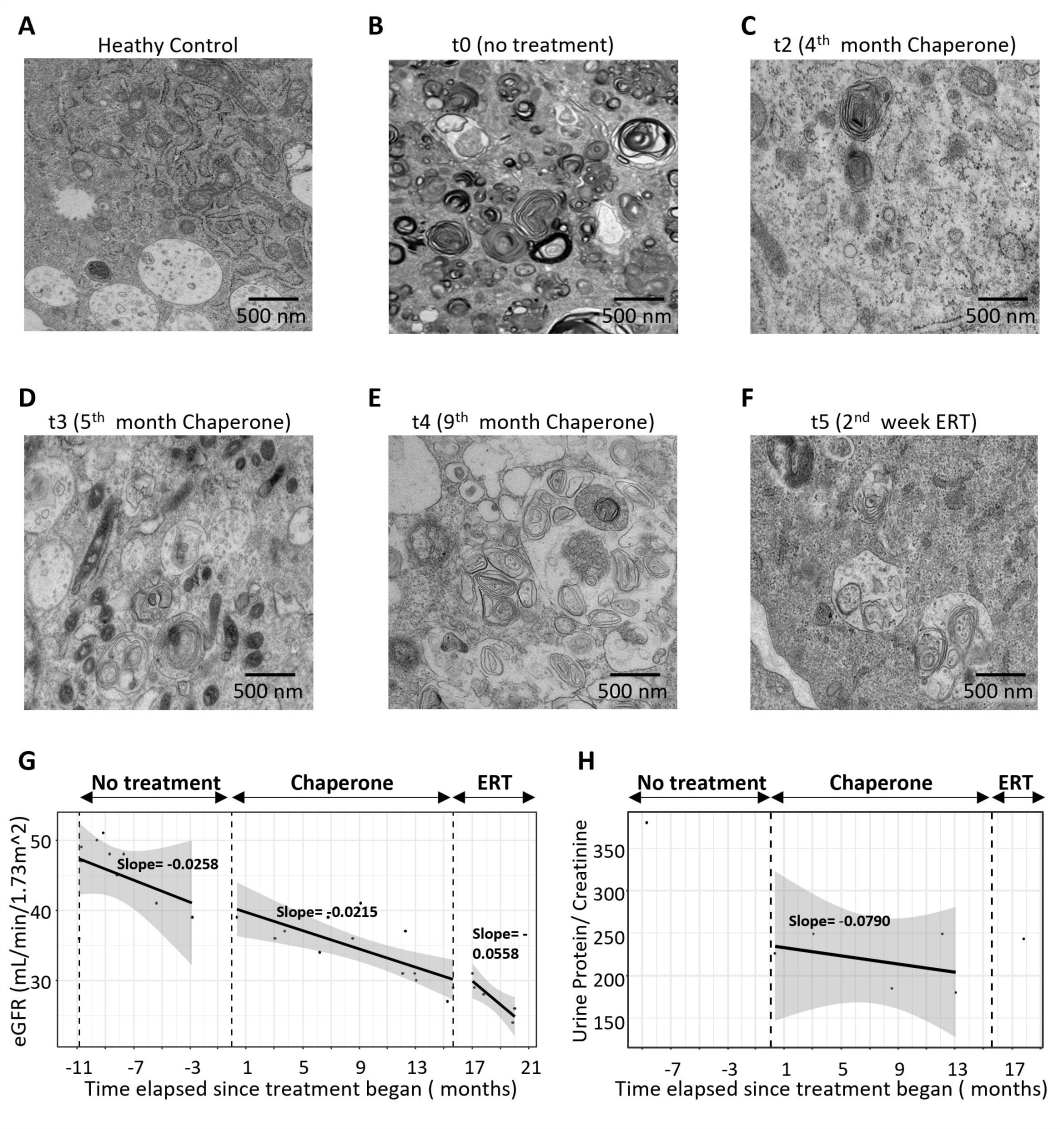
Temporal changes in kidney function markers and ultrastructural analysis before and after treatment in a Fabry patient. Panels A–F show TEM images highlighting zebra bodies, the lysosomal inclusions characteristic of Fabry disease. (**A**) Healthy control with no detectable zebra bodies. (**B**) Baseline (t0, no treatment). (**C**) 4th month of chaperone treatment (**t2**). (**D**) 5th month of chaperone treatment (**t3**). (**E**) 9th month of chaperone treatment (**t4**). (**F**) 2nd week of enzyme replacement therapy (ERT, **t5**). (**G**) estimated glomerular filtration rate (eGFR) plot showing a progressive decline in eGFR across three phases: pre-treatment, chaperone treatment, and ERT. (**H**) Urine protein/creatinine ratio plot demonstrating a reduction during chaperone treatment. In panels G and H, regression lines represent the rate of decline, shaded regions indicate the 95% confidence interval, and vertical dashed lines mark treatment transitions.

Zebra bodies were evident in TEM images at each time point during treatment (Figure 4B–F). There was an appreciable reduction in the observed density of zebra bodies following treatment initiation. Linear regression analyses suggest a trend of progressive eGFR decline across three phases: pre-treatment, chaperone treatment, and ERT. The rate of eGFR decline was more pronounced before treatment, slowed during chaperone therapy, and accelerated again after the transition to ERT, as indicated by the slopes of the fitted lines in each phase (Figure 4G). The urine protein/creatinine ratio also improved during chaperone treatment (Figure 4H).

**Figure 5:**
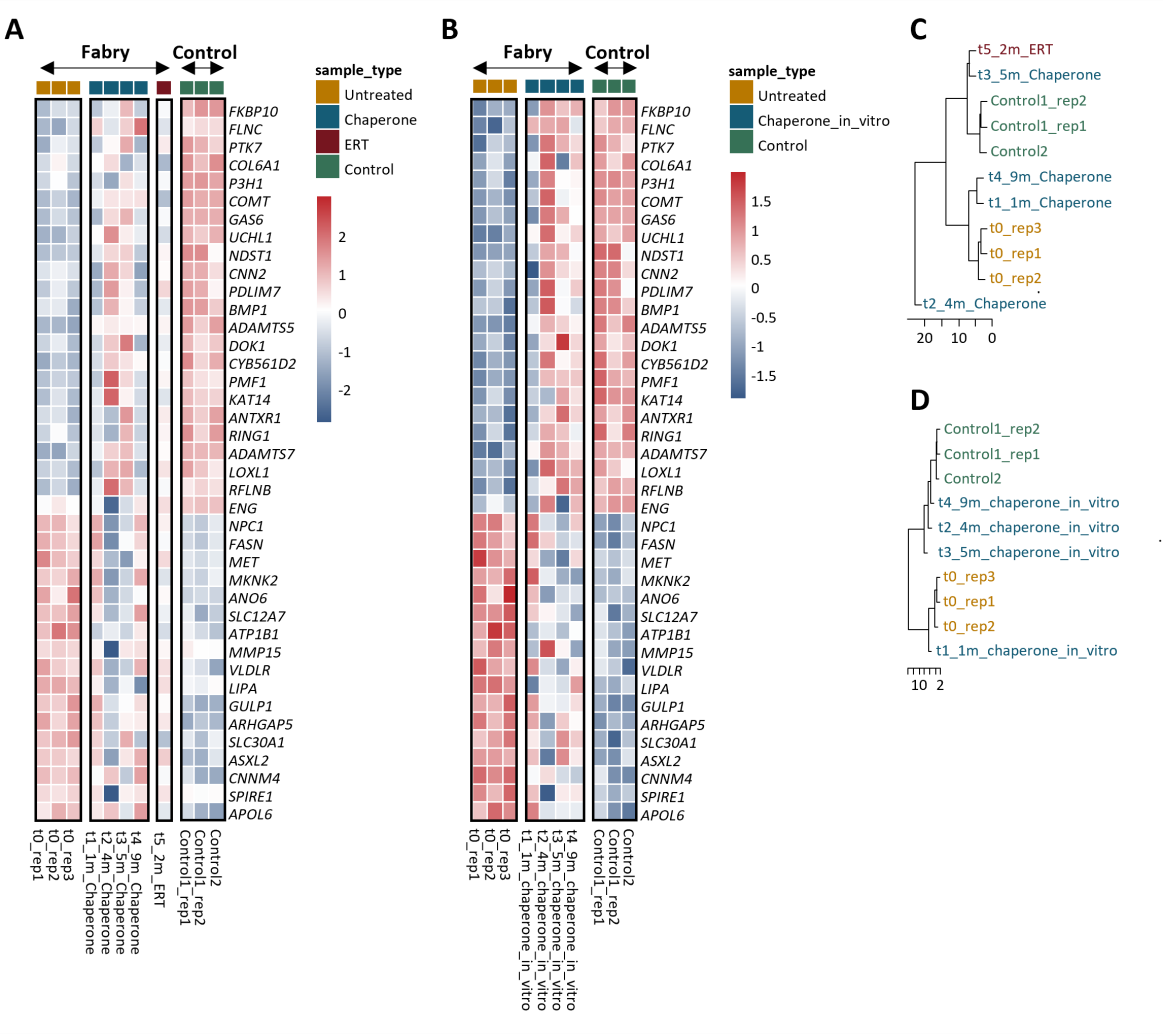
Gene expression changes over time following chaperone treatment and transition to enzyme replacement therapy (ERT) in the Fabry patient. The heatmap in (**A**) shows gene expression profiles over time following chaperone treatment and its transition to ERT, focusing on the most relevant genes identified as differentially expressed in the comparison between untreated Fabry patients and controls. The heatmap in (**B**) illustrates the impact of *in vitro* chaperone treatment on primary hUREC cultures as different time points, using the same genes as in A. The cluster dendrogram in (**C**) visualizes the similarity between samples based on the gene expression data from A, while the dendrogram in (**D**) visualizes the similarity between samples based on the expression data from (**B**). The height of the branches represents the dissimilarity between the gene expression profiles.

Our temporal analysis revealed distinct shifts in gene expression: one month after chaperone treatment, gene expression remains similar to untreated controls, indicating minimal early changes; four months after chaperone treatment, a significant shift occurs, with up-regulated genes decreasing and down-regulated genes increasing, signaling a strong response to treatment; five months after chaperone treatment, the profile begins to resemble untreated controls, suggesting stabilization or recovery; but nine months after chaperone treatment, the profile reverts to a pre-treatment state, suggesting diminishing treatment effects over time. Two months after the switch to ERT, the profile shifts again, moving closer to a control-like state, reflecting adaptation to the new therapy (Figure 5A and C). Also, *in vitro* treatment of primary hUREC cultures with the chaperone at different time points progressively shifts the gene expression profile of Fabry samples closer to controls (Figure 5B and D).

In an attempt to combine the patient’s clinical timeline with the transcriptomic datasets derived from patient hURECs, we developed a ‘Similarity Score’. This metric yields a single, interpretable value per sample that quantifies the degree of transcriptional similarity to the healthy control profile, relative to baseline. The score is derived from z-score normalized gene expression values and reflects gene-level deviations in treated samples relative to both reference states. By aggregating across genes, it facilitates an objective assessment of treatment-induced transcriptomic changes (Supplementary Figure S5). We anticipate that, following further validation with additional Fabry nephropathy patients, this scoring approach could serve as a real-time indicator of therapeutic success in future clinical studies of Fabry nephropathy patients.

## Discussion

Fabry disease is a lysosomal storage disorder that is characterized by the accumulation of glycosphingolipids, notably GL-3, in many tissues, including the kidneys. Molecular pathways involving lipid metabolism, ECM remodeling, and cellular stress response are critical to the disease pathogenesis in Fabry disease [51,52]. The overall pathology relates to the accumulation of GL-3 in cells and tissues of the body, giving a wide spectrum of disease phenotypes that cause diagnostic confusion and delay [53–55].

Typically, Fabry nephropathy presents with the detection of proteinuria in the second or third decades of life [56]. This may often prompt a kidney biopsy which may show glomerular lesions such as glomerular sclerosis and wider kidney involvement, including tubular atrophy and interstitial fibrosis [57]. The decline in kidney function is usually at a comparable rate to diabetic nephropathy. Analyses of Fabry Registry data show that of the patients aged ≥40 years, 41% of males were in CKD stages 3–5 (eGFR <60 ml/min per 1.73 m^2^) [21].

In addition to specific treatments for Fabry disease, where there is evidence of Fabry nephropathy, proteinuria should be treated with antiproteinuric therapy, using angiotensin-converting enzyme (ACE) inhibitors or angiotensin receptor antagonists. The role for sodium-dependent glucose cotransporter 2 (SGLT2) inhibitors in Fabry disease has not yet been explored, but clinical studies are underway [58].

However,if assessed earlier in life, kidney involvement secondary to Fabry disease may be evident. The renal damage is thought to be secondary to GL-3 deposition, and treatment with ERT can achieve clearance of deposits [59]. As demonstrated by this case, later-stage kidney involvement may not be reversible. Early diagnosis and early initiation of treatment are therefore likely to have the greatest impact on kidney outcomes.

Reduction or normalization of plasma GL-3 and reduction in urinary GL-3 excretion can be achieved, and monitoring of these disease biomarkers can be useful. Recent data show that the analysis of lyso GL-3 (globotriaosylsphingosine) is preferred to GL-3 with regard to Fabry disease [60]. In order to evaluate the kidney involvement, the practical and commonly used biomarkers are serum creatinine and proteinuria. Here, we demonstrate that hURECs can be used for both diagnosis, given their directly comparable results under TEM to a native kidney biopsy, and investigating transcriptomic disease signatures and response to treatment. The use of hURECs is obviously limited to research settings at present, but there may be a precedent to bring this into clinical practice. As discussed, the use of urinary microscopy to identify Mulberry cells has largely been phased out, except in certain countries such as Japan.

The patient we have described had a *GLA* missense variant in the catalytically active region [61]. This change in polarity may affect how the residue interacts with other molecules in the ER, reducing the enzyme’s ability to traffic to the lysosomes. Up to 50% of Fabry disease patients carry a Galafold™-amenable *GLA* variant, and as next-generation sequencing approaches, including gene panels, whole-exome, and whole-genome sequencing, continue to become more commonplace, it is likely that more patients with *GLA* variants will be identified earlier, allowing more patients to benefit from the chaperone therapy if indicated or ERT, leading to improved patient outcomes.

As hURECs are non-invasive to collect, and GL-3 is known to accumulate in renal epithelial cells [62], we used hURECs and then TEM to determine if a histological diagnosis was feasible and whether the amount or burden of zebra bodies correlated with biochemical kidney phenotypes (such as eGFR and proteinuria). hURECs can be difficult to isolate and culture, due to the limited number present in the urine, and their short lifespan [63]. The dark inclusions in the lysosomes are zebra bodies, and it was possible to determine a reduction in GL-3 accumulation after periods of treatment. Galafold™ has previously been shown to cause a reduction in GL-3 in renal cells [64]. Clinical trials have indicated a statistically significant reduction in sphingolipid inclusions after one year of treatment with Galafold™ [65].

Transcriptomic analysis of hURECs from the patient with Fabry disease revealed 551 significantly up-regulated genes and 347 significantly down-regulated genes compared with a healthy control. We observed an increase in *NPC1* expression, a critical cholesterol transport gene linked to lysosomal function. The increase in *MMP15* expression is consistent with the role of matrix metalloproteinases in ECM turnover and fibrosis in Fabry nephropathy [66]. Conversely, genes crucial for ECM integrity and maintenance, including *ADAMTS5, LOXL1, COL1A1,* and *COL6A2,* were all down-regulated in the patient hURECs, indicating dysregulated ECM dynamics, as previously observed in Fabry disease [51]. Our pathway analysis revealed dysregulation in lipid homeostasis, ER stress response, and ECM organization, all processes involved in the Fabry nephropathy disease progression. These findings highlight the transcriptional perturbations in the renal pathology of Fabry disease.

Chaperone therapy and ERT are important treatment modalities in Fabry disease, yet their impact on kidney health remains underexplored. Here, we monitor the transcriptional disease signature in the kidney using patient hURECs over the course of the disease progression and the response to different therapies. During chaperone therapy, we observed an initial reversal in the disease pattern, marked by a reduction in up-regulated disease-associated genes, including *NPC1, MMP15,* and *VNN1,* as well as a restoration in the expression of ECM-related gene expression. However, after nine months, the transcriptional profiles shift towards the pre-treatment states, correlated with the decreasing kidney function and reduction in eGFR. Following the switch to ERT, the transcriptional profile stabilizes with reduced expression of stress and lipid metabolism-related genes. These findings suggest that although the chaperone therapy provided an initial transient benefit, ERT may offer a more consistent and sustained amelioration of the disease-associated pathways in the kidney. This analysis highlights the value of longitudinal and organ-specific investigations to optimize therapeutic strategies. The use of hURECs is valuable in this context due to its non-invasive nature, providing effective insights into treatment response and efficacy in the clinical management of Fabry disease. Previous studies have shown that differentially methylated DNA regions, as well as various other epigenetic modifications in Fabry disease patients, may be responsible for the pathophysiology and phenotypic heterogeneity in Fabry disease [67]. In future studies, hURECs could be used for both transcriptomic and epigenetic analyses, such as methylation assays, to further define the role of epigenetics in Fabry disease pathophysiology and to develop novel Fabry disease treatments targeting epigenetic modifications.

We treated hURECs *in vitro* directly with Migalastat to validate its direct efficacy in correcting disease-associated molecular signatures. Notably, treatment with Migalastat induced a reversal of these pathological signatures, as evidenced by RNA-seq data, shifting them toward wildtype expression levels. This approach demonstrates that direct treatment of hURECs with Migalastat provides a robust platform to evaluate the amenability of other missense mutations to pharmacological chaperones and to screen novel chaperone molecules using this *in vitro* methodology.

Recent advances in the treatment and cure of another LSD, Metachromatic Leukodystrophy (MLD) [68], may pave the way to a cure for Fabry disease. MLD is an autosomal recessive disorder, and affected patients rapidly progress after symptom onset, with a loss of motor and neurocognitive function, which is fatal [69]. MLD is caused by an accumulation of sulfatides in the lysosome due to a defective arylsulfatase A (ARSA) enzyme due to variants in *ARSA*. A revolutionary gene therapy, Libmeldy™, has been approved by the European Medicines Agency [70] as a cure for MLD. Libmeldy™ utilizes a lentiviral vector encoding the wildtype *ARSA* cDNA which is transfected into the patient’s own hematopoietic and progenitor stem cell (HSPC) population and transfused back into the patient, forcing *ARSA* expression in the HSPCs and all progeny of these cells [69]. This effectively cures patients of the disease if administered in the pre-symptomatic stages. As an LSD caused by a dysfunctional single enzyme, it is possible that this technology could also be applied to Fabry disease, eventually providing a cure. Again, as we have shown the power of utilizing hURECs to determine Fabry disease therapies, we propose that hURECs could be used as a real-time indicator of treatment success. Indeed, using a scoring approach, which, as we have shown, quantifies the transcriptomic profiling of hURECs, offers a valuable quantitative measure for monitoring efficacy in future Fabry nephropathy therapy trials.

The underlying disease mechanisms of Fabry nephropathy are shared between males and females, including the accumulation of GL-3 and associated cellular dysfunction. However, the clinical phenotype in females is often more variable due to random X chromosome inactivation, which can lead to a mosaic pattern of enzyme activity and disease expression in different tissues. This variability can influence both the severity and progression of renal involvement in heterozygous females. Despite this, we believe that the transcriptomic profiling approach we have described in this study is equally applicable to females with Fabry nephropathy. The method captures molecular deviations at the cellular level, regardless of the underlying X chromosome inactivation pattern and, therefore, has the potential to serve as a robust indicator of treatment response in both sexes.

## Concluding remarks

Fabry disease is a rare X-linked disorder that can be diagnostically challenging and lead to late presentations. Diagnosis relies on clinical, biochemical, histological, and genetic testing.

In order to determine renal involvement, hURECs could be a useful diagnostic tool in place of a kidney biopsy, as the detection of zebra bodies under TEM is readily achievable. In addition, the RNA-seq data derived from cultured hURECs, once validated, could be used to generate a disease signature for Fabry disease, which could be used diagnostically and as a more accurate marker for response to therapy than GL-3 levels and zebra body quantification, and to determine the effectiveness of novel treatments for Fabry disease nephropathy.

**Clinical Perspectives**

- Fabry disease is a rare X-linked lysosomal storage disorder which often presents late and remains difficult both to diagnose and to monitor the efficacy of treatments.
- Our study demonstrates the utility of human urine-derived renal epithelial cells (hURECs) to diagnoses Fabry nephropathy and to provide a disease signature using transcriptomics which can be used as a real-time readout of treatment efficacy and response.
- hURECs are a valuable non-invasive tool to diagnosis and monitor the treatment of Fabry disease, and implementing this into clinical use should be a priority.

## Supplementary material

Online supplementary figure 1

Online supplementary figure 2

Online supplementary figure 3

Online supplementary figure 4

Online supplementary figure 5

Online supplementary table 1

Uncited online supplementary material 1

## Data Availability

The gene quantification data for all samples, including expression values for each gene across the genome, are publicly available and can be accessed at https://doi.org/10.6084/m9.figshare.28310816 Due to the presence of patient-sensitive genetic information, the raw sequencing data and alignment files cannot be made publicly available. However, these data are available upon reasonable request to the corresponding author. Supplementary tables, containing the full results of the differential expression analysis (Table S1) and enrichment analysis (Table S2), can be accessed as part of the supplementary materials. All in-house scripts used for data processing and analysis are available in the GitHub repository at https://github.com/juearcilaga/Fabry_hUREC_Transcriptomics/tree/main.

## Abbreviations

ARSA: arylsulfatase A
CKD: chronic kidney disease
CPM: counts per million
ECM: extracellular matrix
EM: electron microscopy
ER: endoplasmic reticulum
ERT: enzyme replacement therapy
FBS: Fetal Bovine Serum
HSPC: hematopoietic and progenitor stem cell
LFSE: log fold chance error rate
LSD: Lysosomal storage disorders
MLD: Metachromatic Leukodystrophy
NES: normalized enrichment score
REGM: renal epithelium cell growth medium
RNA-seq: RNA sequencing
TEM: transmission electron microscopy
eGFR: estimated glomerular filtration rate
hURECs: human urine-derived renal epithelial cells
log2FC: log2 fold change
padj: adjusted p-value
