# Supplementary figures and images for: Urine-derived renal epithelial cells for deep phenotyping and transcriptomic response to therapy in Fabry disease
Source: Clin Sci (Lond). 2025 Jul 28;139(14):791–808. doi: 10.1042/CS20255570 (PMC12409995; doi:10.1042/CS20255570)

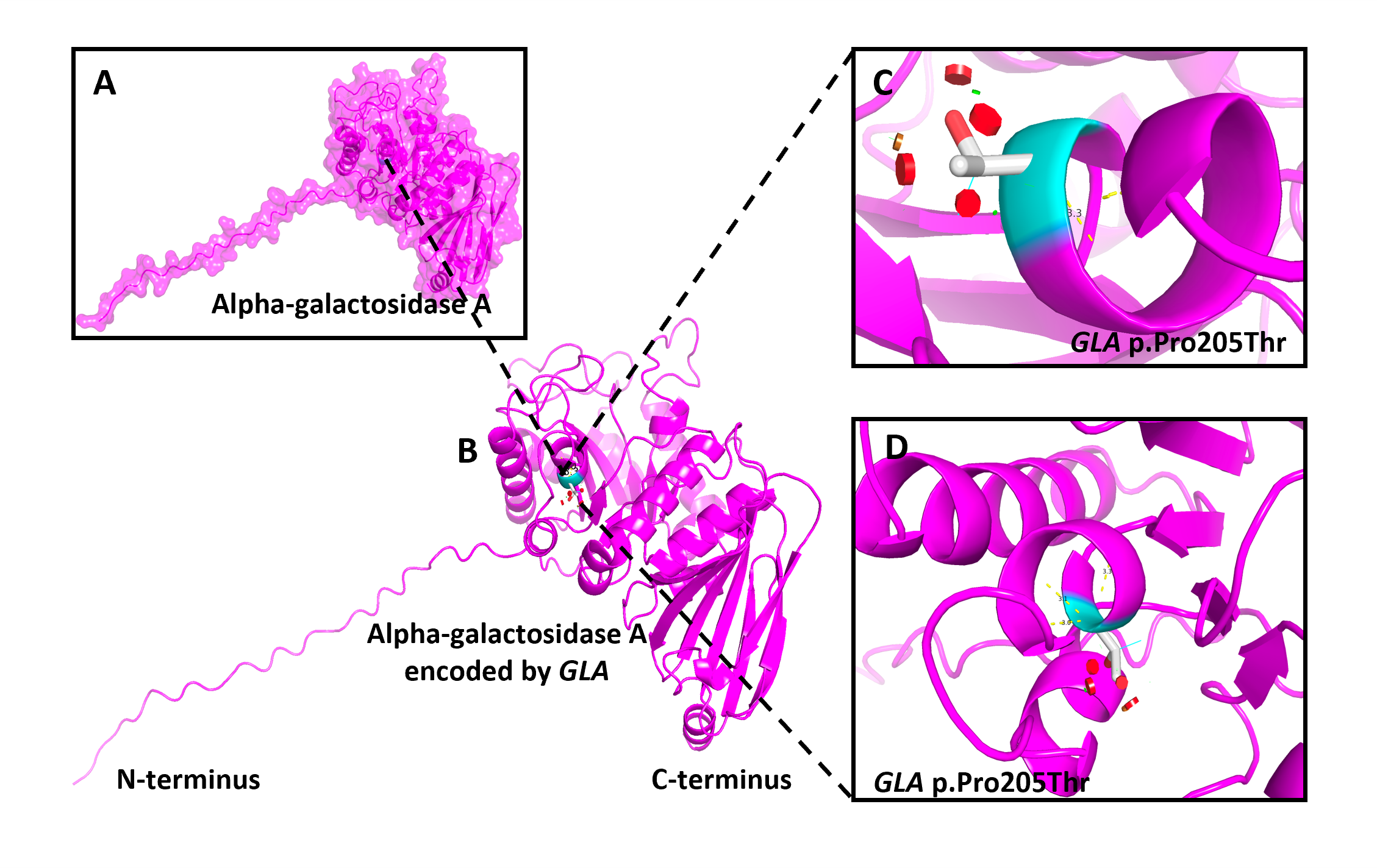

Supplement: Online supplementary figure 1 [file CS-139-14-CS20255570-s001.tif]

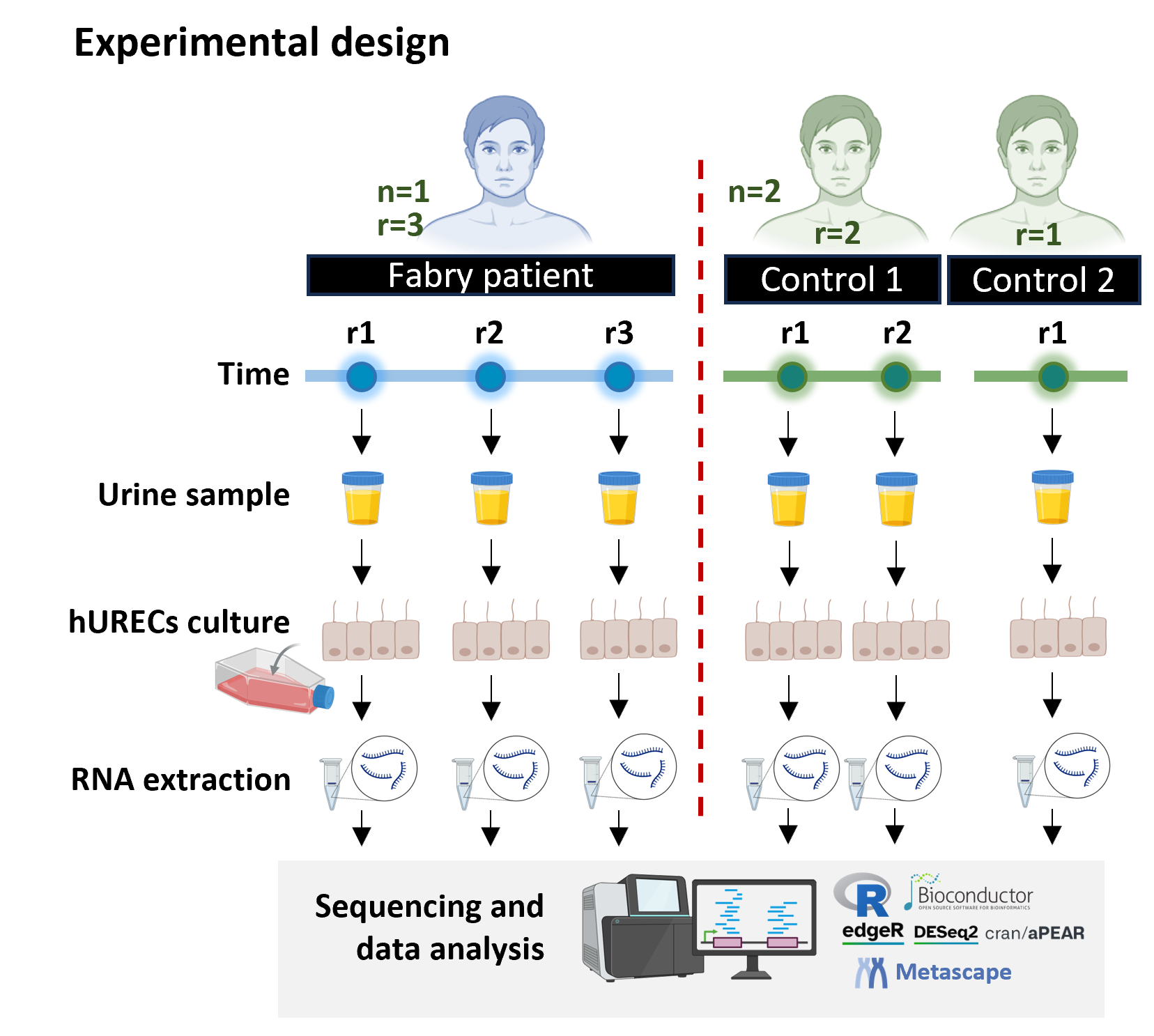

Supplement: Online supplementary figure 2 [file CS-139-14-CS20255570-s002.tif]

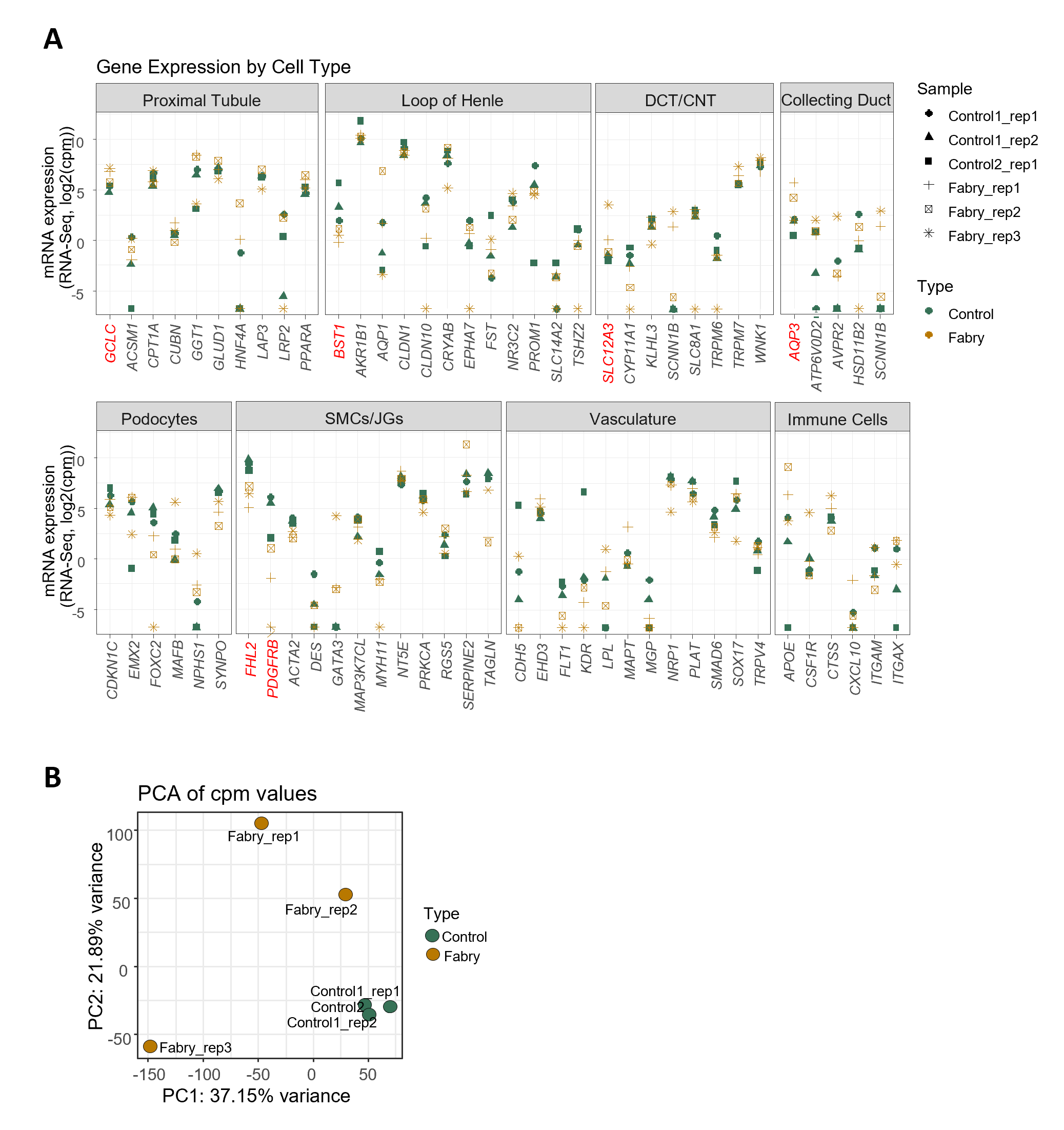

Supplement: Online supplementary figure 3 [file CS-139-14-CS20255570-s003.tif]

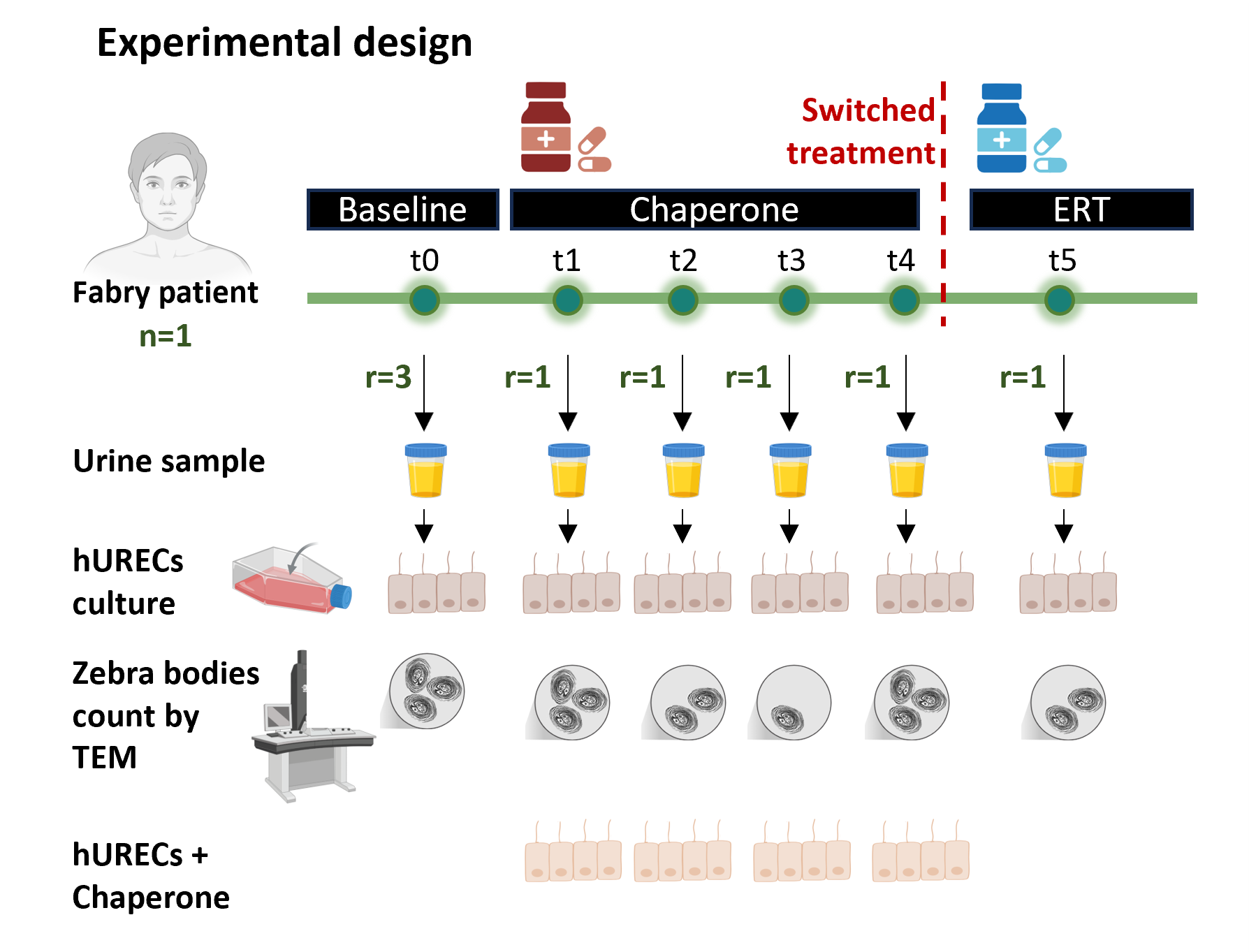

Supplement: Online supplementary figure 4 [file CS-139-14-CS20255570-s004.tif]

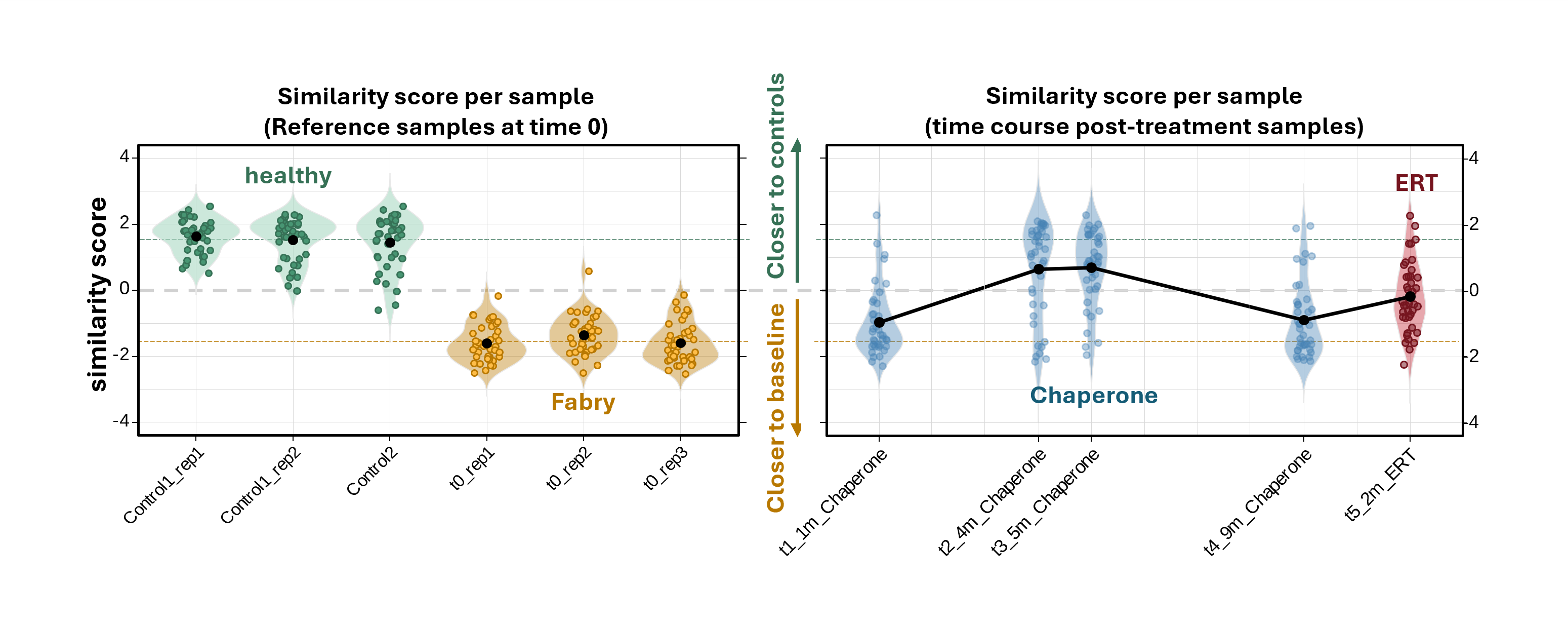

Supplement: Online supplementary figure 5 [file CS-139-14-CS20255570-s005.tif]
