# Supplementary material for: Urine-derived renal epithelial cells for deep phenotyping and transcriptomic response to therapy in Fabry disease
Source: Clin Sci (Lond). 2025 Jul 28;139(14):791–808. doi: 10.1042/CS20255570 (PMC12409995; doi:10.1042/CS20255570)
Supplement: Uncited online supplementary material 1 [file CS-139-14-CS20255570-s007.docx]

**Supplementary Information**

**Urine-derived renal epithelial cells for deep phenotyping and transcriptomic response to therapy in Fabry disease**

Praveen Dhondurao Sudhindar^1^, Sarah Orr^1^, Eve Miller-Hodges^2^, Katrina M. Wood^3^, Shalabh Srivastava^1,4^, Colin G. Miles^1^, Holly Mabillard ^1^, Zachary T. Sentell^5^, Marco Trevisan-Herraz^5^, Juliana E. Arcila-Galvis^5^, and John A. Sayer^5,6,7^

**SUPPLEMENTARY FIGURE LEGENDS.**

**Figure S1. Structural modeling of the missense variant p.Pro205Thr in human alpha-galactosidase A. (A)** The predicted 3-dimentional structural model of human Alpha-galactosidase A, encoded by *GLA* on chromosome X, generated using AlphaFold Protein Structure Database ([https://alphafold.ebi.ac.uk](https://alphafold.ebi.ac.uk/)) and UniProtKB (<https://www.uniprot.org/uniprot/>) with associated codes: AF-P06280-F1v4 and P06280 respectively.  **(B)** Position of missense SNV c.613C>A p.Pro205Thr modelled. **(C)** Perspective 1: Missense SNV c.613C>A p.Pro205Thr modelled with the most probable (83.3.% likelihood) rotamer demonstrated (cyan). **(D)** Perspective 2: Missense SNV c.613C>A p.Pro205Thr modelled with the most probable (83.3.% likelihood) rotamer demonstrated (cyan).

**Figure S2. Experimental design of the transcriptomic analysis of hURECs from Fabry patient and healthy individuals.** The study involves one Fabry patient (n = 1) and two healthy individuals (n = 2. Urine samples were collected at three different time points (r1, r2 and r3) for Fabry and at two different time points for Control 1 (r1 and r2). Human urinary renal epithelial cells (hURECs) were cultured from the urine samples, followed by RNA extraction. Extracted RNA underwent sequencing, and the data were analyzed using bioinformatics tools, including edgeR, DESeq2, and Metascape, within the R Bioconductor framework.

**Figure S3. Cell type markers gene expression and Principal Component Analysis from Fabry patient and healthy individuals.**  **(A)** Expression levels of cell type marker genes across samples. CPM (counts per million) values for marker genes associated with specific cell types were plotted. The marker genes were selected based on their relevance to cell type identification as reported in Balzer et al., 2022 and Garcia et al., 2022. Genes shown in red font are evidently differentially expressed **(B)** Principal Component Analysis (PCA) plot of gene expression data (log_2_(CPM+1)).

**Figure S4. Experimental design of the transcriptomic analysis of hURECs for temporal follow-up of treatment effects**. Urine samples were collected from a Fabry patient (n = 1) at six-time points: **t0** (baseline, no treatment; three replicates, r = 3), **t1–t4** (during chaperone treatment; single replicate, r = 1), and **t5** (transition to enzyme replacement therapy, ERT; single replicate, r = 1). hURECs were cultured from each urine sample and analysed using transmission electron microscopy (TEM) and RNA sequencing. For transcriptomic analysis, cells were split into two conditions: untreated and treated with chaperone in culture. Sequencing data were analysed using edgeR, DESeq2, and Metascape within the R Bioconductor framework to explore transcriptomic changes across treatments and conditions.

**Figure S5. Quantification of transcriptional response to treatment in Fabry hUREC.** This score enables the objective and interpretable tracking of transcriptional rescue in response to therapy. The y-axis represents the Similarity Score, where positive values reflect greater similarity to the healthy (control) transcriptome, and negative values indicate closer resemblance to the Fabry disease (baseline) state. The central black line connects the sample-level similarity scores, which are calculated as the average (black dots) of the gene-level similarity scores for each sample. Violin plots display the distribution of gene-level scores within each sample. Left panel: Reference samples include healthy controls (green) and untreated Fabry samples (yellow), illustrating a clear separation between control and disease states. Right panel: Post-treatment samples demonstrate dynamic shifts in transcriptional similarity over time. Chaperone-treated samples (blue) exhibit a progressive shift toward the control profile, with the most substantial improvement observed at t2 and t3, followed by a relapse at t4. The T5 time point, corresponding to the first sampling after switching to ERT (red), still shows a transcription profile more similar to the baseline, but indicating a partial shift toward the control state.

**SUPPLEMENTARY TABLE LEGENDS:**

**Supplementary Table 1. Results of differential gene expression analysis between Fabry patient and healthy individuals**. Only genes with statistically significant expression changes (adjusted p-value < 0.05) and an absolute log2 fold change (|log2FoldChange|) greater than 1 are included. The columns provide the following information: Gene.name (gene symbol), baseMean (average normalized expression across all samples), log2FoldChange (log2-transformed fold change between conditions), lfcSE (standard error of log2FoldChange), stat (test statistic), pvalue (raw p-value), padj (p-value adjusted using Benjamini-Hochberg correction), ensembl (Ensembl gene ID), and Entrez (Entrez gene ID).

**Supplementary Table 2. Results of gene set enrichment analysis (GSEA) for gene ontology (GO) Biological Process terms.** Only terms with an adjusted p-value (padj) < 0.05 are included. The columns provide the following information: ID (GO term identifier), Description (name of the Biological Process GO term), setSize (number of genes in the gene set), enrichmentScore (GSEA enrichment score for the gene set), NES (normalized enrichment score), pvalue (raw p-value for the GO term), and p.adjust (p-value adjusted for multiple testing using the Benjamini-Hochberg correction). Positive NES values indicate enrichment in upregulated genes in Fabry, while negative NES values indicate enrichment in downregulated genes.
